# *Arabidopsis* DNA topoisomerase I alpha is required for adaptive response to light and flower development

**DOI:** 10.1242/bio.024422

**Published:** 2017-05-11

**Authors:** Evgenia V. Kupriyanova, Evgeniy V. Albert, Aleksandra I. Bliznina, Polina O. Mamoshina, Tatiana A. Ezhova

**Affiliations:** Department of Genetics, Faculty of Biology, Lomonosov Moscow State University, 119234, Leninskiye Gory 1/12, Moscow 119234, Russia

**Keywords:** DNA topoisomerase, Light response, Shade avoidance, Floral morphogenesis, RNA sequencing

## Abstract

DNA topoisomerase I alpha (TOP1α) plays a specific role in *Arabidopsis thaliana* development and is required for stem cell regulation in shoot and floral meristems. Recently, a new role independent of meristem functioning has been described for TOP1α, namely flowering time regulation. The same feature had been detected by us earlier for *fas5*, a mutant allele of *TOP1α*. In this study we clarify the effects of *fas5* on bolting initiation and analyze the molecular basis of its role on flowering time regulation. We show that *fas5* mutation leads to a constitutive shade avoidance syndrome, accompanied by leaf hyponasty, petiole elongation, lighter leaf color and early bolting. Other alleles of *TOP1α* demonstrate the same shade avoidance response. RNA sequencing confirmed the activation of shade avoidance gene pathways in *fas5* mutant plants*.* It also revealed the repression of many genes controlling floral meristem identity and organ morphogenesis. Our research further expands the knowledge of TOP1α function in plant development and reveals that besides stem cell maintenance TOP1α plays an important new role in regulating the adaptive plant response to light stimulus and flower development.

## Abstract (precis)

**Summary:** This study expands upon the existing knowledge of *Arabidopsis* DNA topoisomerase gene *TOP1α* function in plant development and demonstrates its important new role in regulating shade response and flower development.

## INTRODUCTION

DNA topoisomerases function to relieve torsional stress in the DNA helix by introducing transient breaks into the DNA molecule. These enzymes regulate genome stability and processes of DNA recombination, repair, replication and transcription. In *Arabidopsis thaliana* (hereafter *Arabidopsis*) topoisomerase I alpha (TOP1α) has recently been shown to regulate nucleosome density or positioning at regulatory gene regions, thus probably allowing transcription factors to bind to their target genes (Liu et al., 2014). Genetic studies show strong synergistic interactions between *top1α* alleles and mutations in *Arabidopsis* genes encoding Polycomb Group Protein (PcG) subunits, which suggest that TOP1α is required for PcG-mediated repression of gene expression (Graf et al., 2010; Liu et al., 2014). Overrepresentation of PcG targets among genes whose expression is altered in the *top1α-2* mutant suggests that *TOP1α* affects PcG-mediated epigenetic regulation in *Arabidopsis* plants (Liu et al., 2014). These findings explain the specific developmental function of TOP1α, which has been actively studied.

To date several mutations in *TOP1α* have been described in *Arabidopsis*. The detailed study of *mgo1-1*, *mgo1-4*, *top1α-1*, *top1α-2* and *fas5* mutants showed that all of them display defects in specific developmental processes related to the function of apical shoot and floral meristems. In accordance, their main phenotypic features are an enlarged apical meristem and stem fasciation (Laufs et al., 1998; Takahashi et al., 2002; Graf et al., 2010; Liu et al., 2014; Albert et al., 2015a).

Unlike *clv* mutants, which also demonstrate larger meristems and fasciation of the inflorescence stem, mutations in *TOP1α* cause a continuous fragmentation of the shoot apex into multiple meristems, which leads to the formation of extra branches. Meristem enlargement in *top1* and *clv* mutants are associated with an ectopic expression of stem cell maintenance gene *WUS*. However, in contrast to *clv* mutants, which maintained enlarged stem cell pool in apical and floral meristem throughout their life cycle, *TOP1α* mutants gradually lost the ability to maintain stem cells during development. The cells of the inflorescence apical meristem (IM) increased in size and lost the indeterminate state (Albert et al., 2015a). The progress of development is provided by ectopic formation of new meristems (Laufs et al., 1998; Albert et al., 2015a).

In addition to these conspicuous features, mutations in *TOP1α* cause other developmental changes. Some of them are related to meristem malfunction. Extra carpel development, for instance, is explained by the reduction of *AG* binding to *WUS*, which results in a prolonged *WUS* expression, and a consequent loss of floral determinacy (Graf et al., 2010; Liu et al., 2014). The relation between other features in *TOP1α* mutants and meristem functioning is less evident. Among these features we note the accelerated transition to the reproductive phase, and a delay in floral development, both described for the *fas5* mutant (Albert et al., 2015b). Moreover, we demonstrated that the *fas5* induced an accelerated bolting in *lfy-10* mutant that had slightly delayed flowering on Dijon-M (Dj) background (wild type for *fas5*), but fully transformed flowers into branched vegetative structures in *fas5 lfy-10* plants (Albert et al., 2015b). The *fas5* also accelerated inflorescence characters in flowers of *ap1-1*, *ap1-20* and *ap2-1* mutants thus indicating that *TOP1α* could play important role in determining floral meristem development via mediating the expression levels of floral meristem identity genes (Albert et al., 2015b). Recently, an early flowering phenotype was demonstrated for *top1α-10* and *mgo1-7* mutants (Gong et al., 2017).

To clarify seemingly opposite effects of *fas5* on bolting initiation and flower development, we studied the *fas5* plant development in long day condition and performed RNA sequencing (RNA-seq) to investigate the molecular basis of *TOP1α* role in phase transition and flowering time.

## RESULTS

### Mutations in *TOP1α* leads to constitutive shade avoidance syndrome

**Fig. 1.**
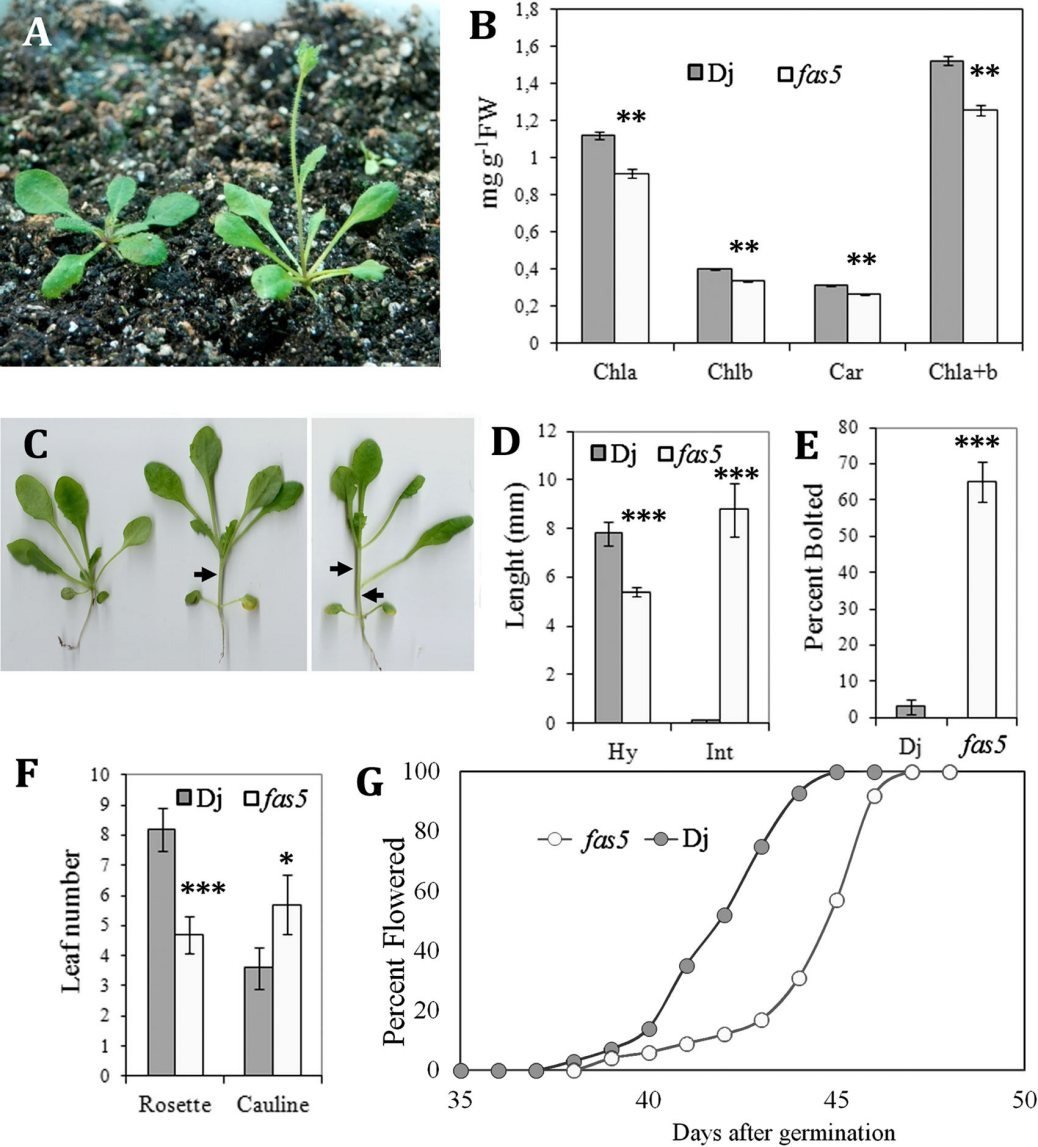
**Morphological features of LD *fas5* and Dj plants.** (A) Dj (left) and *fas5* (right) plants at the age of 4 weeks; *fas5* has elongated petioles, elevated leaf angles and develops inflorescence. (B) Chlorophyll (Chl) and carotenoid (Car) content in *fas5* is lower than in Dj; values are the means of three biological replicates±s.d. (C) Dj (left) and two *fas5* (right) 3-week-old plants after transition to low light for 3 days demonstrate SAS; arrows indicate elongated internodes in *fas5*. (D) Hypocotyl and first internode length in plants after 3 days in low light; mean of two biological replicates±s.d., *n*=25 plants per genotype. (E) Percentage of 4-week-old plants with inflorescences larger than 1 cm; mean of two biological replicates±s.d., *n*=50 plants per genotype. (F) Rosette (Ros) and cauline (Cal) leaf number. (G) Dynamics of flower opening (percent of plants with open flowers); the data represent one experiment, *n*=75 plants per genotype. Similar results are obtained in two independent experiments. Asterisks represent statistically significant differences of the mutant relative to wild-type plants (*P<0.05; **P<0.005; ***P<0.001 in Student's *t*-test).

Plants homozygous for the *fas5* mutant allele were grown under inductive photoperiods and compared with the wild-type Dj plants. Young mutant plants demonstrate slightly elevated leaf angles (hyponasty), elongated petioles and lighter leaf color associated with a slightly reduced chlorophyll and carotenoid level (Fig. 1A,B). The combination of these changes indicates that mutant plants display the shade avoidance phenotype (Franklin, 2008; Casal, 2012; Roig-Villanova and Martínez-García, 2016). This phenotype is detected in the ‘long days’ mutant plants in both the growth room (Fig. 1A) and glasshouse (Fig. S1A), but disappears in the growth chamber under higher light intensity.

After transferring the long days plants that had been grown in the growth room under 130 µmol·m^−2^ s^−1^, to the conditions of reduced light intensity (60 µmol m^−2^ s^−1^) for 3 days, both Dj and *fas5* show the shade avoidance syndrome (SAS). In these three days Dj plants lift up the leaves and demonstrate fast hypocotyl elongation. Under the same conditions, the length of *fas5* hypocotyls remains unchanged, while the length of rosette internodes greatly increases (Fig. 1C,D), thus resembling the phenotype of the double mutant *phyA phyB* (Devlin et al., 1996).

Mutant plants bolt earlier (Fig. 1A,E) and appear with fewer rosette leaves (Fig. 1F) than the wild-type plants, thus confirming the previously reported data for *fas5* plants growing dynamics in a glasshouse (Albert et al., 2015b). This feature of *fas5* is independent of light intensity. Under all conditions *fas5* demonstrates earlier transition to the reproductive stage than Dj, although the exact time of inflorescence development varies. Despite early inflorescence initiation, the first open flower in *fas5* plants developed later and after forming more cauline leaves (Fig. 1F,G). Consequently, after quick transition to the reproductive stage, *fas5* mutant reduce the pace of development. The main features of flower morphology in *fas5* were described by us earlier (Albert et al., 2015b).

**Fig. 2.**
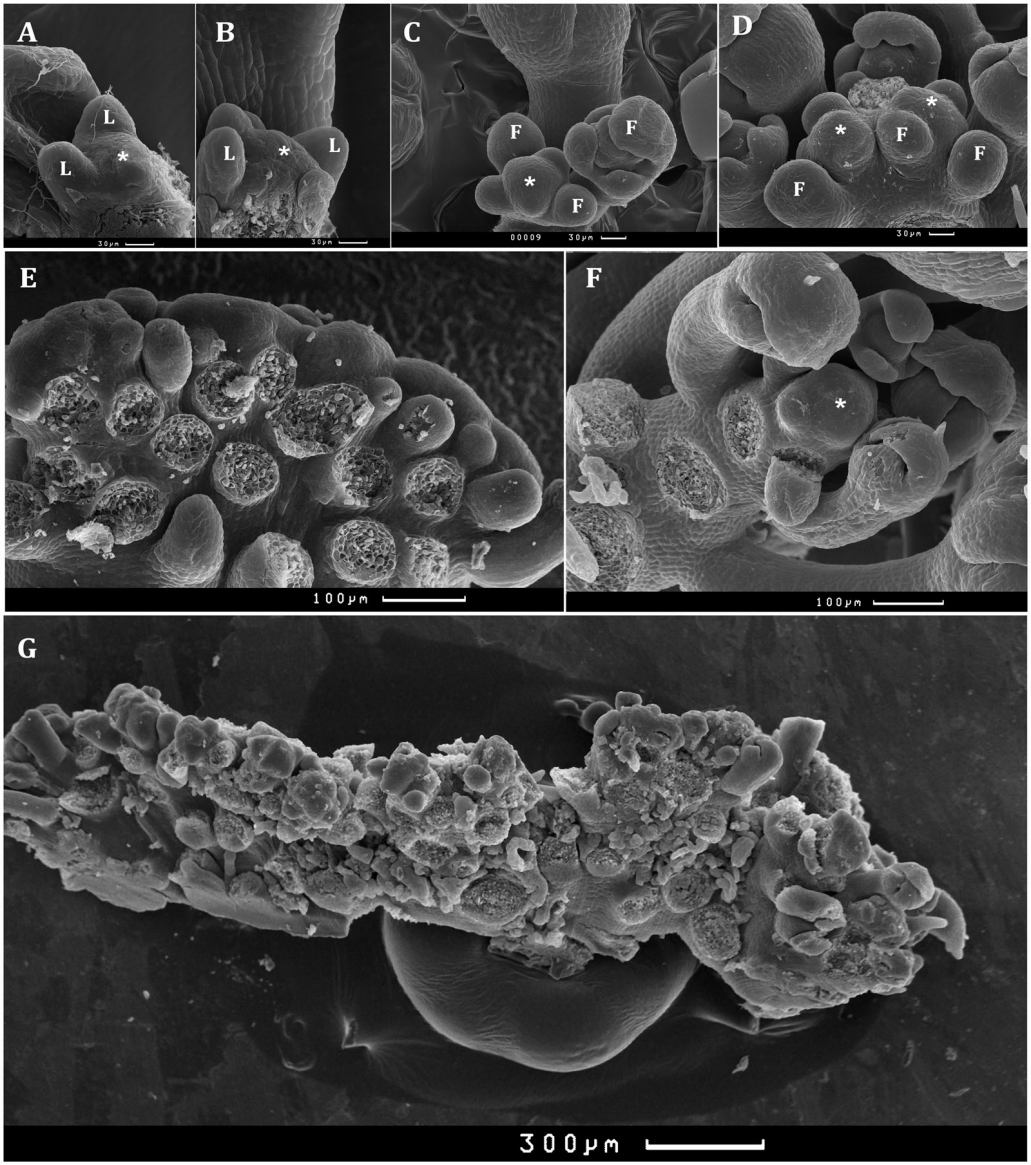
**Scanning electron microscopy of apical inflorescence meristems (IM) in LD plants.** (A,B) Shoot apical meristem of one-week-old Dj and *fas5* plants, respectively. (C,D) IM of Dj and *fas5* plants, respectively, at the flowering initiation phase; IM of *fas5* fragmentizes into two parts. (E-G) IM of *fas5* mature plants with weak (E) and strong (G) fasciation from the glasshouse and from the growth chamber (F). L, leaf primordium; F, floral primordium; *, IM. Scale bars: 10 μm in A-D; 100 μm in E-F; and 300 μm in G.

Unexpectedly, light intensity has great influence on IM morphology and related degree of stem fasciation. Mutant plants growing in the growth room demonstrate apical meristem elongation after formation of several leaves (Fig. 2A,B) and fragments into multiple meristems (Fig. 2C-E). As the plant matured, these abnormalities became progressively more severe (Fig. 2C-E,G) and also strictly depended on growth conditions. At the end of the life cycle, IM in the growth room and glasshouse frequently looked like a meristem comb (Fig. 2G) and the stem usually terminated in a mass of carpelloid tissue (Fig. S1B), as in strong *lfy* mutants (Weigel et al., 1992). The percentage of such plants in the glasshouse was greater (up to 67%) than in the growth room (10%). In the growth chamber, IM maintains its integrity (Fig. 2F) and formation of carpelloid structures is not observed.

Thus, although the expressivity of *fas5* depends on growth conditions, we conclude that *TOP1ɑ* mutation shortens specifically the vegetative phase, although it also slightly delays the shoot apical meristem to IM transition. Considering that the expressivity of *fas5* features depends on light intensity, we assume that the *fas5* mutation affects light perception or response.

**Fig. 3.**
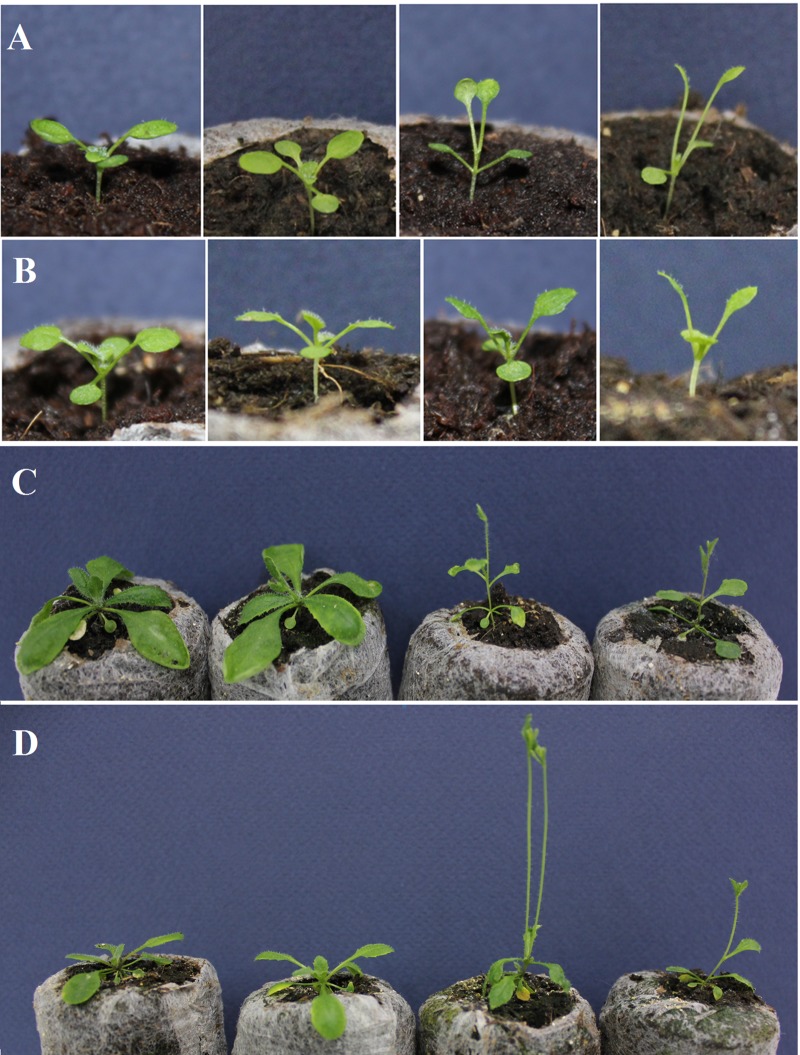
**Morphological features of *top1α-1* and *top1α -*2 and wild-type plants.** 10-day-old seedlings (A,B) and 19-day-old plants (C,D) of Ler and *top1α-2* (two left and two right plants, shown in A and C), and Col and *top1α-1* (two left and two right plants represented in B and D).

Other alleles also show constitutive shade avoidance response under long days. A strongest reaction is detected in *top1ɑ-2* (Ler background), which demonstrates leaf hyponasty from the early seedling stage (Fig. 3A). The *TOP1α-1* (Col wild type) shows weaker constitutive SAS (Fig. 3B), while *mgo1-7* has intermediate phenotype (data not shown). After transferring the plants from 130 µmol m^−2^ s^−1^ to the reduced light intensity (60 µmol m^−2^ s^−1^) for 3 days, occasional *top1ɑ-1* plants increase their rosette internodes while most *top1ɑ-2* (Fig. S2) and more than half *mgo1-7* studied plants do demonstrate this specific feature of phytochrome deficiency.

All previously studied mutant alleles of *TOP1ɑ* demonstrate the accelerated transition to the reproductive phase in our experiments (Fig. 3C,D). All of them have reduced rosette leaf number and are characterized by an early bolting (Fig. S3). These observations provide evidence that phenotypic features revealed in *fas5* mutant are common for other allelic mutants, though they differ by the expressivity of constitutive SAS.

### Mutation in *TOP1α* leads to a deeper and more extensive activation of genes, than to suppression

We performed RNA-seq to determine the effects of the *fas5* mutation on genome-wide gene expression in the apices of young inflorescences. This analysis shows that mutation in *TOP1ɑ* causes changes in the expression level of 3901 genes: 2300 of the differentially expressed genes (DEGs) present higher expression in the mutant plants (Table S2), and 1601 present lower expression (Table S3). For the majority of genes only a slight change of the expression level is observed. If we omit DEGs with a log_2_-fold change (FC) less than one, the number of DEGs falls to 550 for downregulated and 1389 for upregulated genes.

**Fig. 4.**
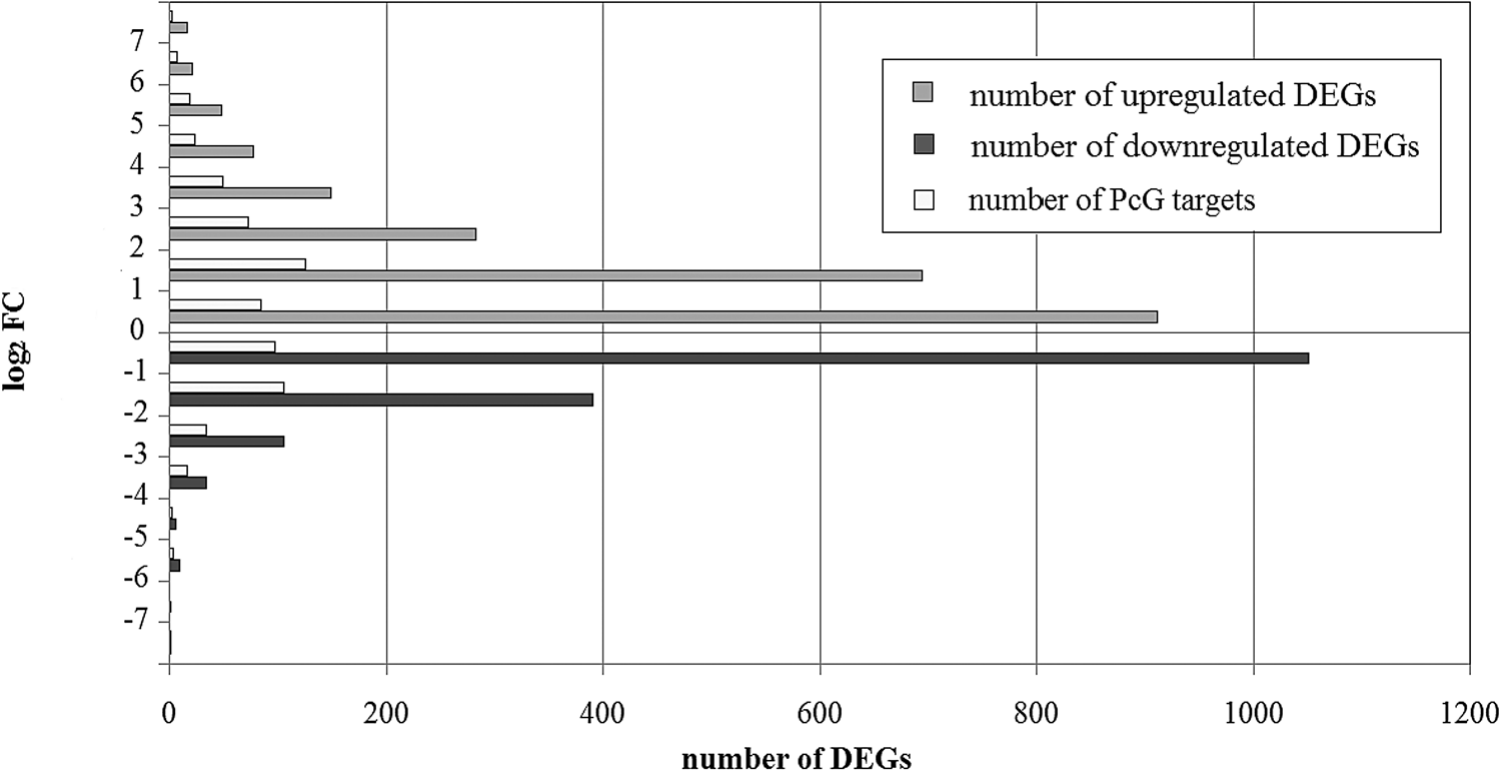
**DEGs distribution according to expression level.**

There is about 70% of genes with a less than twofold difference in the log_2_FC-expression level in the group of activated genes, and 90% among repressed genes. The fraction of genes with a log_2_FC between 2 and 4 is twice as high among activated DEGs (18.8%) as among repressed genes (8.7%). The number of DEGs with a more than fourfold difference in the log_2_FC expression level is about 7% among activated genes, but only about 1% among repressed genes. Thus, the mutation in *TOP1ɑ* leads to a deeper and more extensive activation of genes, than to suppression (Fig. 4).

It has been shown recently that genes with H3K27me3 in their chromatin preferentially represent PcG targets and require *TOP1ɑ* for expression (Liu et al., 2014). We compare the list of *fas5* DEGs with the list of H3K27me3 target genes in *A. thaliana* (Zhang et al., 2007). We find 16% and 18% of potential PcG target genes among the total list of repressed and activated genes in *fas5* (Tables S4 and S5). These values are close to the total fraction of genes with H3K27me3 in the whole genome (Liu et al., 2014). However, a portion of potential PcG targets among DEGs in *fas5* depends on FC in their expression level (Fig. 4). When considering the DEGs with less than onefold differences in log_2_FC, a portion of PcG targets is 9% for both upregulated and downregulated DEGs, but this portion increases in the group of DEGs with a greater elevation in log_2_FC. The largest number of PcG targets is found among activated DEGs with a log_2_FC between 5 and 6 (40%) and repressed genes with a log_2_FC between 3 and 4 (47%). Thus, our RNAseq result confirms the previously reported microarray data demonstrating a strong enrichment of PcG targets among DEGs in the *top1α-2* mutant (Liu et al., 2014).

To carry out a more detailed analysis of biological processes impaired in *fas5*, we characterize DEGs using the Functional Annotation Clustering Tool implemented in DAVID Bioinformatics Resources 6.7 (https://david-d.ncifcrf.gov/) to get DEGs clusters according to their functional similarity (Huang et al., 2007).

Between the ten annotation clusters with an enrichment score higher than threefold and a false discovery rate (FDR) of ≤0.05 one can see that activated DEGs in *fas5* fall into two major annotation clusters with maximum enrichment score values associated with chloroplast, chloroplast parts, and plastid (Table S6). Enrichment in terms related to cell wall, external encapsulating structure (cluster 3), response to organic, hormone and endogenous stimulus (cluster 4), extracellular region and glycoprotein (cluster 5), organic and fatty acids biosynthetic/metabolic process (cluster 6), response to inorganic substance and some others is also observed.

The majority of repressed genes fall into annotation cluster 1, which is associated with DNA binding, nucleus, regulation of transcription, transcription factor (regulator) activity, regulation of RNA metabolic process and so on (Table S7). Annotation cluster 2 is enriched in terms that are involved in chromatin and chromosome organization, histone fold and acetylation. Small annotation clusters 6 and 8 are also associated with acetylation, and histones H4 and H2A (Table S7). Several clusters (3, 4, 5, 7, 10) are enriched in terms associated with development (regionalization, pattern specification and xylem and phloem pattern formation, flower development and differentiation, meristem development, stem cell maintenance and shoot development).

Overall, our analysis revealed significant qualitative differences between the annotation clusters of activated and repressed DEGs. Among activated genes, those associated with chloroplasts prevail, but the regulators of transcription and development are dominating among repressed genes. Since most processes associated with chloroplasts depend on light stimulus, we suggest that genes regulated by light should be present among activated DEGs.

To obtain a comprehensive list of genes involved in light response and developmental processes among DEGs, we analyze Gene Ontologies for term enrichment using the AgriGO Single Enrichment Analysis tool. The full result of this analysis is represented in the Supplementary Information (Tables S8 and S9). This analysis confirms the results from DAVID. Among activated DEGs in *fas5*, a group of 267 genes (approximately 12% of activated DEGs) are associated with the term ‘plastids’ and 246 genes (11%) are associated with the term ‘chloroplast’. A group of 70 genes is related to response to light stimulus (Table S8, Fig. S4). Downregulated DEGs are enriched in genes involved in developmental processes (154 genes). This group includes the genes associated with flower (58 genes), shoot (43 genes), meristem (24 genes), root (28 genes), and leaf (23 genes) development (Table S9, Fig. S5). AgriGO also detected genes associated with the terms ‘response to light stimulus’ (42 genes) and ‘photoperiodism’ (9 genes, Table S9). Genes related to flowering, light response and photoperiodism are of particular interest. DEGs with an absolute value of FC≥2 and log_2_FC≥1 will be considered below.

### Mutation in *TOP1α* alters an expression level of key genes involved in light response and shade avoidance

**Fig. 5.**
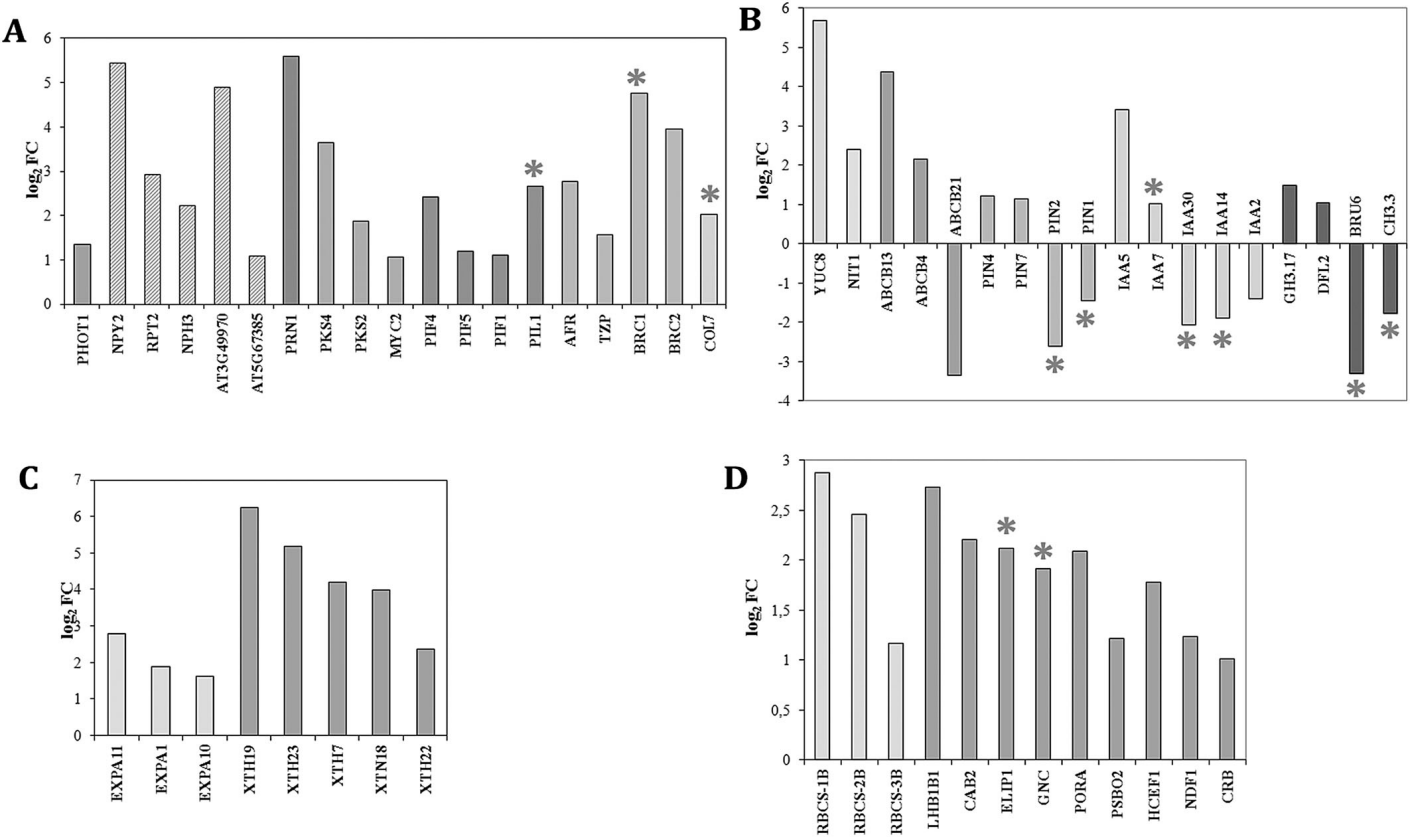
**DEGs involved in light response and SAS.** (A) BL and R:FR light signaling genes. (B) Auxin synthesis, transport, and response genes. (C) Auxin-regulated genes involved in cell loosening and expansion. (D) Genes regulating chloroplast development and functioning. PcG-target genes are marked with asterisks.

We focus on genes associated with different aspects of light perception and shade response and reveal four interconnected groups of genes showing increased expression in *fas5*. The first group contains 19 genes (Fig. 5A) including: *PHOT1* encoding a blue light (BL) photoreceptor phototropin, five paralogues *NPY2*, *RPT2*, *NPH3/RPT3*, *At3G49970*, and *At5g67385* encoding BTB/POZ domain-containing proteins that might act in fine-tuning the photoreceptor PHOT1 activity, *PRN1* involved in BL signaling, and *MYC2* (encodes a transcription factor), a regulator of BL-mediated photomorphogenic growth and blue-light and far-red-light regulated gene expression.

Several light-response DEGs are involved in phytochrome-mediated photomorphogenesis. Five of them encode bHLH phytochrome-interacting transcription factors, PIFs (*PIF1*, *PIF4*, and *PIF5*) or a *PIF-*like factor (*PIL1*), which participate in the phytochrome B signaling pathway. The genes *PKS1* and *PKS2* encode phytochrome kinase substrate (PKS) family proteins, which are involved in phyA and phyB and BL signaling. We also include in the first group the *AFR* gene, a part of the phyA-mediated signaling transduction pathway, the *TZP* gene that plays a critical role in phyB signaling. The genes *BRC1* and *BRC2* also increase their expression level in *fas5*. These genes contribute to reduced branching under shade and are negatively regulated by phytochrome (Aguilar-Martínez et al., 2007; González-Grandío et al., 2013). The gene *COL7* is also involved in branching regulation and acts as an enhancer of the shade avoidance response via promotion the expression of *PIL1* mRNA in response to shade (Wang et al., 2013).

The second group (Fig. 5B) contains seven upregulated genes involved in auxin synthesis (*YUC8*), transport (*ABCB4*, and *ABCB13* genes encoding the ATP-binding cassette B family of transporter proteins, and *PIN4* and *PIN7* genes for auxin efflux transmembrane transporters) and auxin response (*IAA5* and *IAA7*). The third group contains auxin-regulated genes of expansins and xyloglucan endotransglycosylase/hydrolases (Fig. 5C) playing an important role in loosening and extension of plant cell walls (Cosgrove, 2016). These genes provide rapid organ elongation during adaptive plant growth (Sasidharan et al., 2008; Casal, 2012). Among DFGs in *fas5* with increased expression we find eight genes: three genes of expansins (*EXPA1*, *EXPA10*, and *EXPA11*), and five genes of xyloglucan endotransglycosylases hydrolases (*XTH7*, *XTH18*, *XTH19*, *XTH22*, and *XTH23*). These three groups of genes contain a total of 33 upregulated genes that are an integral terminal part of adaptive growth responses known as SAS*.*

In addition to these three groups of activated genes we reveal light-regulated genes in *fas5* with elevated expression involved in chloroplast development and functioning (Fig. 5D). Among them are the genes encoding RBCS-1B, RBCS-2B and RBCS-3B (ribulose bisphosphate carboxylase small chain family proteins), CAB2 (chlorophyll A/B binding proteins), LHB1B1 (light-harvesting chlorophyll-protein complex II subunit B1), ELIP1 (an early light-inducible chlorophyll A-B binding family protein), GNC AT5G56860 (GATA transcription factors regulating chlorophyll biosynthesis), PORA (protochlorophyllide oxidoreductase A), PSBO2 (photosystem II subunit O-2), and some other genes. Because PIFs regulate chlorophyll biosynthesis (Huq et al., 2004; Moon et al., 2008; Liu et al., 2013), the observed shift in expression of these genes is not surprising. By adjustment of growth and development plants can optimize light capture for photosynthetic utilization under shade conditions. Therefore, the revealed changes in chloroplast gene expression can also be considered as part of the SAS.

Among repressed light-regulated genes we find three genes controlling auxin transport (*ABCB*, *PIN1*, and *PIN2*) and three genes of Aux/IAA transcriptional factors (*IAA2*, *IAA14*, and *IAA30*) that function as repressors of early auxin response genes (Fig. 5B). Strongly repressed MADS-box gene *MAF1* closely related to the negative regulator of flowering *FLC* is also found among light-regulated genes. This gene is simultaneously present both among light-regulated genes and flowering genes, which will be discussed below.

In addition to light-regulated genes in *fas5*, we find DEGs, which are not regulated by light but may be related to the above-mentioned group of auxin-related genes. Three activated genes are involved in auxin synthesis and homeostasis: they are the nitrilase gene *NIT1*, which regulates auxin biosynthesis from indole-3-acetonitrile, and two auxin-induced GH3 family genes *GH3.17* and *DFL2* controlling auxin homeostasis via the synthesis of auxin conjugates with amino acids. We also find two other GH3 family genes *GH3.3* and *BRU6/GH3.2* among repressed genes in *fas5* (Fig. 5B).

It is important to note that in growth response, which is a final stage of SAS, more plant hormones are involved than just auxin (Martinez-Garcia et al., 2010; Stamm and Kumar, 2010). Among DEGs in *fas5*, we find activated genes related to abscisic acid (68 genes), jasmonic acid (29 genes) and gibberellin stimulus (27 genes) along with 9 repressed genes associated with the gibberellin signaling pathway (Tables S8 and S9). In this article we will not discuss these genes, although signaling pathways of these hormones not only actively interact with the auxin pathway but also regulate expression level and/or activity of PIF and other components of SAS pathway (Bou-Torrent et al., 2014; Leivar and Monte, 2014).

It is interesting that among the considered activated DEGs related to SAS, only 17% belong to potential PcG targets (8 genes out of 48); and vice versa, among downregulated DEGs associated with SAS (all of them regulate auxin associated processes) PcG targets (7 genes) prevail over non-targets (2 genes).

### Mutation in *TOP1α* changes an expression level of many genes involved in flowering initiation and development

**Fig. 6.**
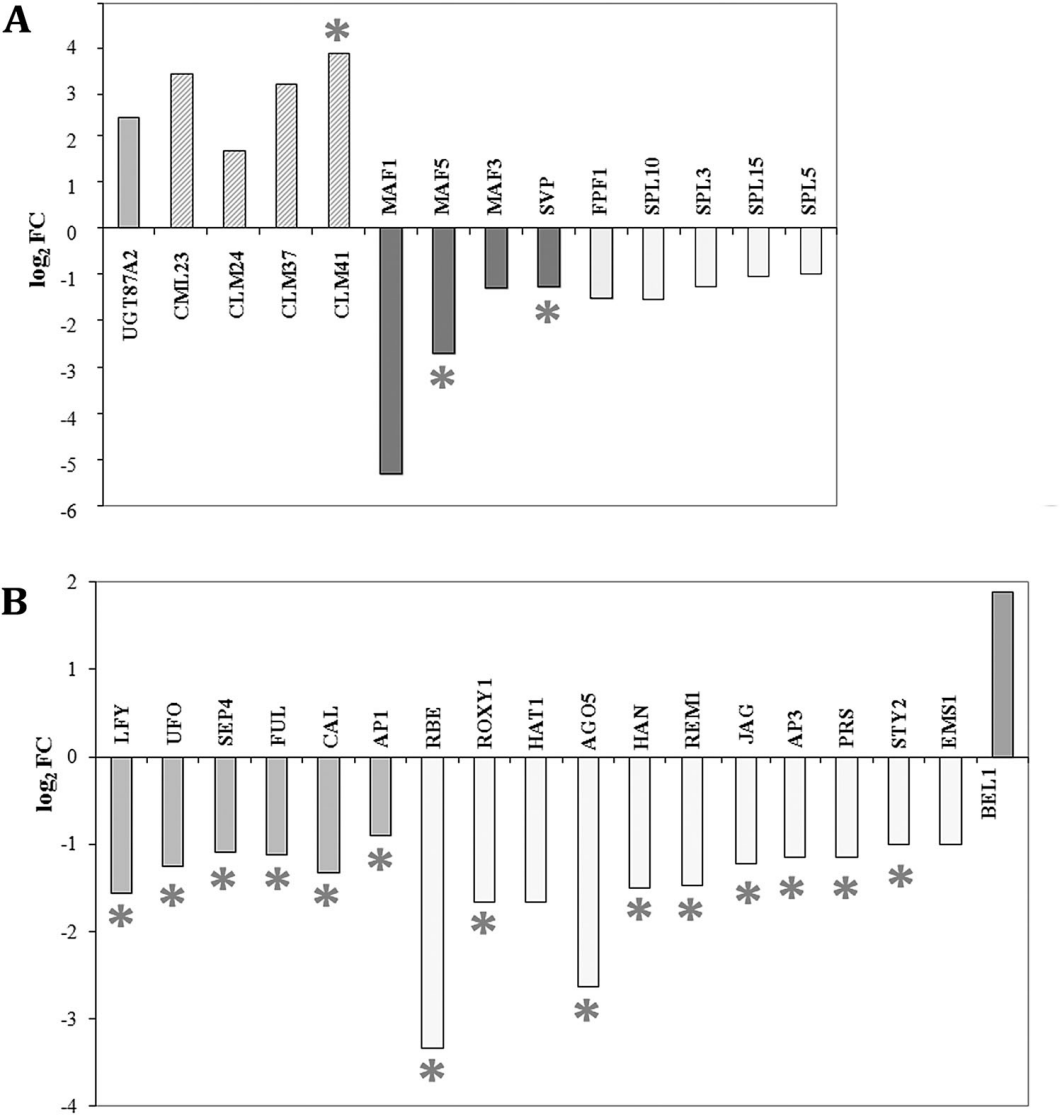
**DEGs involved in flowering.** (A) Flowering time genes. (B) Floral meristem identity genes and floral organ developmental genes. PcG target genes are marked with asterisks.

Among DEGs with an increased expression level in *fas5* we find the *UGT87A2* gene encoding a UDP-glycosyltransferase superfamily protein, which promotes flowering (Wang et al., 2012), and two genes encoding calmodulin-like proteins (*CML23* and *CML24*) playing a role in the flowering transition (Tsai et al., 2007). We find two additional genes belonging to the same family of calmodulin-like genes (*CLM41* and *CLM37*) among activated genes in *fas5*, however, their role in flowering time control is not defined (Fig. 6A).

In the group of downregulated genes associated with flowering we determine 26 genes with more than twofold decreased expression in *fas5* (Fig. 6A,B). One gene from this list (*MAF1*) is mentioned above among repressed light-regulated genes. We also find two additional homologues of the *FLC*-clade among genes associated with flowering, the *MAF3* and *MAF5* genes, and the *SVP* gene, which function as a floral repressor together with members of the *FLC*-clade and encode MADS-domain transcription factors. Decrease in the expression level of these repressor genes as well as increased expression of the four above-mentioned positive regulators of flowering initiation may promote flowering in *fas5* plants.

Most of revealed genes with decreased expression belong to positive flowering regulators. This group includes: four SBP-box genes *SLP* (*SPL3*, *SPL5*, *SPL10* and *SPL15*), which define an endogenous flowering pathway in *A. thaliana*, and the *FPF1* gene, which promotes flowering (Fig. 6A). Several DEGs regulate floral meristem identity and floral organ development (Fig. 6B). Among them are the major flower identity integrator gene *LFY* and its co-activator *UFO*, and MADS-box genes *FUL*, *SEP4*, and *CAL*. The latter gene is closely related to *AP1*, expression of which is downregulated slightly less than twofold. All 15 above-mentioned genes play important roles in flowering initiation and floral meristem identity.

Besides these genes, a group of genes associated with flower development contains 11 downregulated genes controlling different aspects of floral organ morphogenesis (Fig. 6B). The *HAN* gene encodes a GATA-3 type transcription factor with a single zinc finger domain and plays an important role in *Arabidopsis* flower meristem organization. A putative C2H2 zinc finger transcription factor *JAG* controls cell proliferation during organ growth by maintaining tissues in an actively dividing state (Dinneny et al., 2004). The *PRS/WOX3* gene promotes cell proliferation in lateral floral primordium domains and is required for the formation of the margin cells of the first and second whorl organs. The MADS-box gene *AP3* is the main regulator of the second and third whorl organ identity. *RBE* and *ROXY1* are required for the early development of petal primordia and petal morphogenesis. The *STY2/SRS2* and *EMS* genes regulate anther development. The *HAT1* gene is involved in floral meristem determinacy and is important for correct gynoecium and fruit development, and *AGO5* promotes the initiation of megagametogenesis. *REM1* may also play a role in flower development. This gene is required for proper integument development and specification of integument identity (Franco-Zorrilla et al., 2002). The downregulation of these above-listed genes may explain numerous alterations in floral morphogenesis that were described earlier for different mutant alleles of *TOP1α*.

Only one gene (*BEL1*) related to flower development is activated in *fas5* (Fig. 6B). It encodes a homeodomain protein required for ovule identity (Bencivenga et al., 2012). In total, only six genes somehow connected with flowering initiation and development are revealed among activated genes, but 26 important regulatory genes are found among repressed genes. Among 32 considered DEGs associated with flowering, 18 are potential PcG targets (56%). If we consider only the downregulated genes, the proportion of PcG targets is even higher (69%).

## DISCUSSION

Analysis of *Arabidopsis* mutants uncovered an important role of *TOP1α* in specific developmental processes. These mutants are characterized by defects in stem cell homeostasis and phyllotaxy (Laufs et al., 1998; Takahashi et al., 2002; Graf et al., 2010). Here we demonstrate that the *fas5* mutation displays novel features. It causes clear SAS features including accelerated bolting, but slightly delays flower development (Fig. 1). Expressivity of shade-associated characteristics depends on light intensity. Most of them disappeared in high light, although accelerated *fas5* bolting was detected under all studied conditions. Our study of phenotype of *top1α-2*, *top1α -1*, and *mgo1-7* mutants shows that constitutive SAS is a common feature for other allelic mutants (Fig. 3). Most of these alleles are thought to be the null alleles and so their phenotypic similarity is expected. Moreover, the fact that in high light many *fas5* malfunctions become minimal (including apical meristem morphology, Fig. 2F) indicates that the discovered change in sensitivity to light is one of the key *fas5* (and probably of other alleles) characteristics that affect other developmental processes, including stem cell homeostasis.

**Fig. 7.**
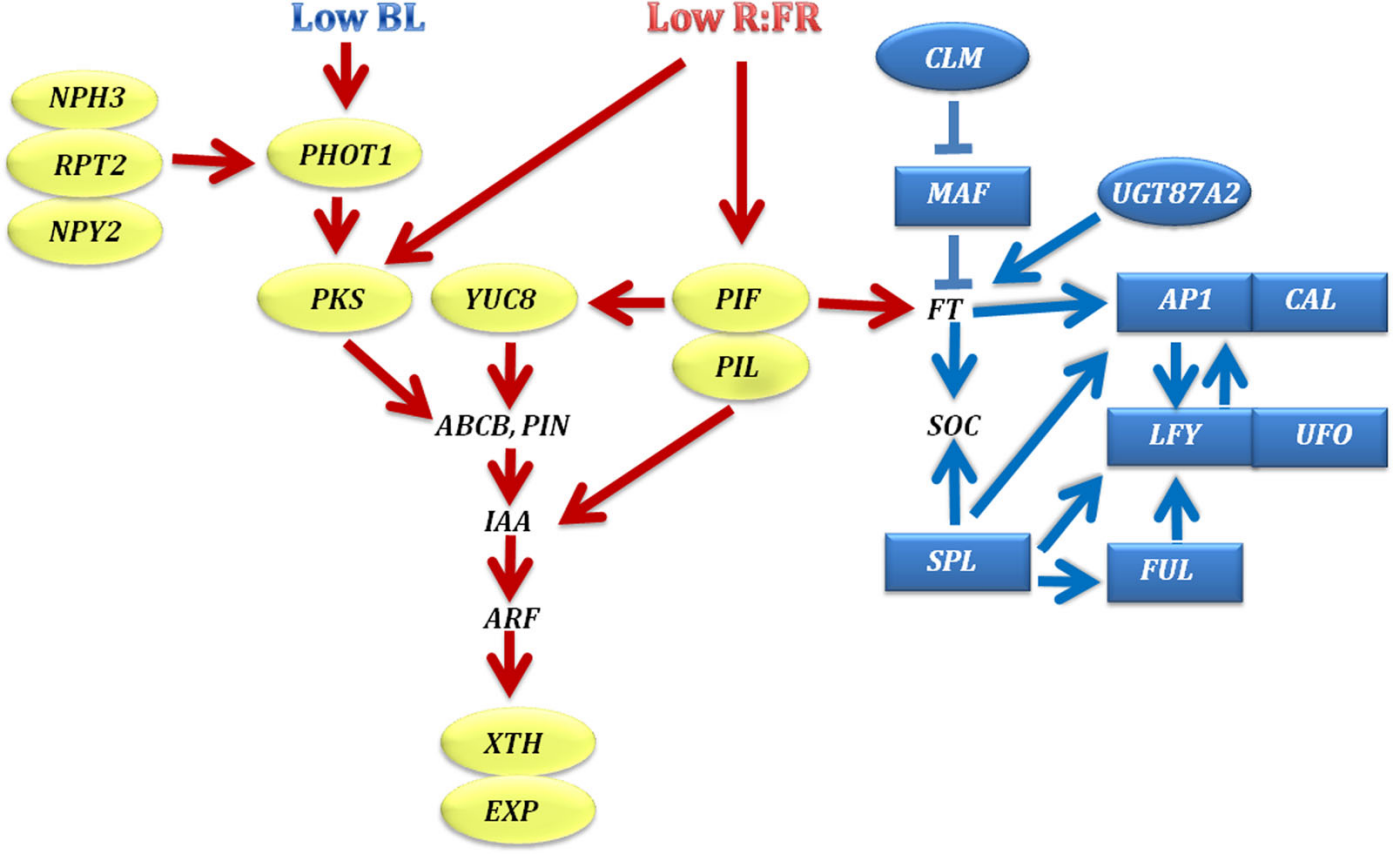
**Mutation *fas5* in the gene *TOP1α* alters expression level of the most key genes regulating shade avoidance (light colored ovals) and flower development (dark colored ovals and rectangles).** Upregulated and downregulated DEGs in *fas5* are shown in ovals and rectangles, correspondingly. Remaining genes either change their expression in different directions (different *ABCB*, *PIN*, *IAA* genes, see Fig. 5B for detail), or aren't revealed among DEGs (*ARF*, *FT*, *SOC*).

SAS is an adaptive growth response activated by a reduced ratio of red to far-red (R:FR) light and reduced BL intensity (Casal, 2012; Pierik and de Wit, 2014; Roig-Villanova and Martínez-García, 2016). One possible explanation for constitutive SAS in *fas5* mutant is an alteration in the gene expression network involved in SAS. RNA-seq confirms an activation of many genes regulating response to low BL and R:FR ratio (Figs 5A and 7). In the *fas5* mutant, we detect upregulation of PHOT1 mRNA. PHOT1 is the primary receptor of BL controlling phototropism, regulating leaf blade expansion, flattening, and positioning under low BL intensity (Ohgishi et al., 2004; Takemiya et al., 2005; Han et al., 2013). We find five activated homologues of *RPT2* and *NPH3/RPT3*. Proteins encoded by *NPY2*, *RPT2*, and *NPH3/RPT3* modify PHOT1 and might act in fine-tuning the photoreceptor activity through modulation of PHOT1 subcellular trafficking and desensitization by degradation (Sakai and Haga, 2012; Liscum et al., 2014).

In *fas5*, we also detect activation of *PKS2* and *PKS4*, important components of the signaling cascade that function as positive regulators of hypocotyl phototropism (Pedmale et al., 2010; Sakai and Haga, 2012; Hohm et al., 2013). Several studies have demonstrated that PKSs serve as a molecular link between phytochrome and phototropin-mediated responses through interaction with phytochromes and PHOT1 (Lariguet et al., 2006; Demarsy et al., 2012; Kami et al., 2014). PKSs may contribute to the phototropic response regulation through modulation of local auxin signaling or transport (Kami et al., 2014). Hence, light can effectively coordinate the activity of major auxin transporters and therefore auxin distribution to control phototropic responses in different organs (Žádníková et al., 2015). The observed changes in the expression of seven auxin transport genes (Fig. 5B) indicate activation of the phototropic response in *fas5* plants, although the opposite changes that we also observe complicate the interpretation.

Several genes activated in *fas5* are involved in phytochrome-mediated photomorphogenesis. We detected activation of phytochrome-interacting factors (PIFs) that play the central role in SAS and optimization of plant development to multiple internal and external signals (Casal, 2012; Leivar and Monte, 2014). In an FR-enriched environment, PIF proteins are stabilized and induce transcription of the *YUC8* gene regulating auxin synthesis (Hornitschek et al., 2012), thus directly linking the perception of a low R:FR signal to changes in free auxin required for shade-induced growth (Li et al., 2012). On the other hand, PIFs regulate transcription of *IAA* genes belonging to the auxin signaling repressor family, which mediate the attenuation of auxin signaling at the illuminated side of plant organs (Sun et al., 2013). In *fas5* we reveal upregulation of *YUC8*, *IAA5*, and *IAA7*. At the same time, *IAA2*, *IAA14*, and *IAA30* are repressed in *fas5* (Fig. 5B). Changes in the mRNA level of all these genes may trigger a transient increase in auxin levels and tissue-specific growth response, which is an integral part of plant tropisms and SAS (de Wit et al., 2015).

Expansins (EXP) and xyloglucan endotransglucosylase/hydrolases (XTH) are two well-characterized cell wall modifying proteins that are implicated in cellular expansion (Cosgrove, 2016). In *fas5* plants activation of five *XTH* and three *EXP* genes are revealed (Fig. 5C). The upregulation of these genes can play an important role in loosening and extension of the plant cell walls during SAS.

Most shade-avoiding plants display reduced branching and enhanced apical growth that help them compete for incident light (Franklin, 2008). Therefore, upregulation of *BRC1* and *BRC2*, which suppress axillary bud outgrowth (González-Grandío et al., 2013), represents an additional evidence in favor of SAS activation in *fas5* (Fig. 5A).

Thus, RNA-seq analysis reveals changes in expression level of key SAS pathway genes. This finding can explain constitutive SAS in *fas5* mutant, including early plant bolting. It was shown that PIF4 and PIF5 transcription factors promote flowering by at least two means: inducing *FT* expression and acting independently of *FT* through an unknown mechanism (Kumar et al., 2012; Thines et al., 2014). *FT* was not revealed among DEGs in *fas5*, since we extracted RNA from inflorescence apices when flowering initiation had already taken place. Nevertheless, *fas5* is characterized by a reduced expression of *SVP* and genes of the *FLC*-clade *MAF1*, *MAF3*, and *MAF5* (Figs 6A and 7). Proteins encoded by these genes may form nuclear MADS-domain complexes (Gu et al., 2013; Mateos et al., 2015). Regulation of flowering time by these complexes appears to be achieved via diverse pathways, including photoperiod or circadian clock, since *MAF1* (Ratcliffe et al., 2003) and *SVP* (Fujiwara et al., 2008; Andrés et al., 2014) participate in the photoperiod pathway. Some indication of *MAF3* involvement in the circadian regulation was also obtained (Mateos et al., 2015). Hence, we cannot exclude that these genes (at least the strongly repressed *MAF1*) are the main component of early flowering in shade and their repression is the main reason for the early phase transition in *fas5* plants. Our datum is in a good agreement with downregulation of *FLC*, *MAF4* and *MAF5* in *top1α-10* seedlings demonstrated by real-time PCR analysis (Gong et al., 2017).

Besides repressed *MAF*s we reveal three activated DEGs, which act early and may promote flowering. *UGT87A2* promotes flowering by repressing *FLC*. An *ugt87a2* mutant exhibited late flowering under both long day (LD) and short day (SD), and its flowering was promoted by vernalization and gibberellin (Wang et al., 2012). Some other activated genes such as genes encoding calmodulin like proteins can promote flowering of *fas5*. The impact of *CML23* and *CML24* in flowering transition via *FLC* repression (Tsai et al., 2007) allows speculating that these genes and probably *CML35* and *CML41* are involved in the repression of close *FLC* homologues *MAF1*, *MAF3* and *MAF5*.

Many other light-regulated genes are upregulated in *fas5*, thus demonstrating essential changes in the light response gene network. These changes together with the SAS phenotype demonstrate that *TOP1*α plays an important role in the regulation of light perception and light response. Apparently an alteration of the initial stages of light perception may explain the activation of the whole set of key SAS genes, most of which are not PcG targets. To identify the primary cause of the constitutive shade response, directed studies of light receptors activity in the *fas5* mutant are required. The similarity between the *fas5* phenotype and the *phyA phyB* double mutant phenotype indicates possible changes in the phytochrome system.

We determine important positive regulators of flowering and floral meristem identity that are repressed in *fas5* (Fig. 6B and 7). Four flower-promoting *SPL* genes (Yamaguchi et al., 2009) are repressed in *fas5*, as well as their targets *FUL*, *LFY* and *AP1*. Repression of the *UFO*, a *LFY* co-regulator, and the *CAL* gene, which functions redundantly with *AP1*, are also detected in *fas5*. These data are in a good agreement with the phenotype of the double mutants *fas5 lfy-10* and *fas5 ap1*, for which a great enhancement of shoot-like features in flowers was described (Albert et al., 2015b). The downregulation of these key positive regulators of floral meristem identity explains delayed flower development in *fas5* mutant.

Decrease in the expression of genes regulating floral organ development can also affect flower morphology (Fig. 6B). For instance, reduced expression of at least three genes *RBE*, *HAN* and *JAG* can explain the reduced number of petals, which is the most easily detected feature in flowers of *fas5* and other allelic mutants. In *fas5* we find 3.3 log_2_FC repression of *RBE*. The effect of this repression is comparable to the loss-of-function *rbe* mutants exhibiting a loss of or aberrant petals (Takeda et al., 2004; Krizek et al., 2006). Flowers of the double mutant *han-2 jag-3* have a reduced number of petals (Ding et al., 2015). A simultaneous decrease in *HAN* and *JAG* expression is exactly what we have seen in the *fas5* transcriptome.

In conclusion, our study explains two seemingly opposite features of the *fas5* mutation in the *Arabidopsis* gene *TOP1α*, i.e. an early transition to the reproductive stage and quick initiation of inflorescence growth on the one hand, and, on the other hand, slowing the pace of flower development (timing of the first flower opening). The first feature is the consequence of the constitutive activation of the shade avoidance gene pathway in *fas5*, which accelerates bolting. We find activation of all key regulators of this pathway including BL photoreceptor phototropin (PHOT1), phytochrome-interacting transcription factors (PIFs and PIL), phytochrome kinase substrate family proteins (PKS) and downstream auxin-dependent components, which adapts plant growth to low BL and low R:FR light conditions. The delayed flower development in *fas5* is the effect of the downregulation of key genes controlling floral meristem identity and floral organ morphogenesis, most of which are PcG targets. The so-called floral integrator gene *LFY*, as well as *FUL*, *SEP4*, *CAL*, *AP3*, *RBE* and many other genes are among them. It is rather doubtful that revealed coordinated changes in the expression level of the whole set of interconnected genes within shade response and floral development networks are random. Given the general role of topoisomerase in maintaining proper DNA topology and its involvement in chromatin remodeling, it is more likely that the *fas5* mutation may cause the direct effect on some of the genes among these networks. The changes in expression level of these target genes, in turn, can affect the expression level of other interacting genes and cause secondary pleiotropic effect on plant morphology. The complete correspondence of the revealed changes in the transcriptome with the phenotype of *fas5* and other allelic mutants leaves no doubt that the gene *TOP1α* is involved in regulation of the shade response gene network.

While we still do not know which of the identified genes with altered expression are the direct targets of topoisomerase activity and what kind of marks do attract TOP1α to these particular genes, answers to these questions is important for elucidation of the possible role of the gene *TOP1α* and other chromatin regulators in the origin and functioning of gene networks which provide coordination between multiple developmental processes and environmental variation.

## MATERIALS AND METHODS

### Plant material and growth conditions

*Arabidopsis thaliana* (L.) Heynh. *fas5* mutant was isolated from an EMS-mutagenized Dijon-M (Dj) seed population and was backcrossed with wild-type Dj plants three times prior to further analysis. The mutation was mapped by whole genome sequencing as described (Leshchiner et al., 2012) using DNA pools of wild-type and *fas5* plants from the F2 *fas5*×Columbia cross. The *fas5* mutation localized to the 9th exon and represented the С to T transition, leading to the replacement of the CAG codon (glutamine 701) by the stop codon TAG. This mutation leads to a premature termination of transcription and loss of a functionally significant C-terminal domain of topoisomerase I (Albert et al., 2014). Plants were grown in a growth room with 130 µmol m^−2^ s^−1^ light exposure at 23°C under long day (LD) conditions (16 h light:8 h dark cycle). All measurements, unless otherwise stated, were carried out in a growth room. Some measurements under LD were performed in a growth room with 60 µmol m^−2^ s^−1^ light exposure, a glasshouse with about 100 µmol m^−2^ s^−1^ light intensity (90-135 µmol m^−2^ s^−1^) and varied day:night temperature, or in a growth chamber with 150 µmol m^−2^ s^−1^ light intensity at 22°C. Plants and apical meristem morphology documentation was performed using light microscopy and scanning electron microscopy as described previously (Albert et al., 2015a). To provide evidence that phenotypic features revealed in *fas5* mutant are not the effect of genetic background, rather they are the cause of the *TOP1α* gene disfunction, phenotype of some other mutant alleles were studied: *top1α-2* on the Ler background and three mutations *top1α -1*, *mgo1-7* on the Columbia (Col) ecotype.

### Pigment assay

Chlorophyll and carotenoid content was measured for 3-week-old plants (before bolting) using three biological repeats. Pigment content was measured spectrophotometrically in acetone extracts of rosette leaves (Lichtenthaler, 1987).

### RNA extraction and sequencing

RNA was isolated from 25- and 20-day-old Dj and *fas5* plants, correspondingly, grown under LD in a growth room. Hand-dissected apices of young inflorescences from 25 wild-type and 25 *fas5* plants were fixed in RNAlater (Qiagen, Germany) in two biological replicates. Total RNA extraction and sequencing of the cDNA libraries were performed in the laboratory of Evolutionary Genomics of Moscow State University using the Illumina Hiseq2000 platform with adaptor ligation and single-end 50 bp reads length.

### Quality control, mapping of RNA-seq reads and bioinformatic analysis

A quality control analysis of raw reads was accomplished by FastQC 0.11.2 (Anders and Huber, 2010). Adapters and low-quality reads were trimmed before data analysis using Trimmomatic 0.32 (Bolger et al., 2014). A total of 150,184,519 reads after trimming were mapped to the *A. thaliana* genome (TAIR10; Table S1) using the BWA package 0.7.1 (Li and Durbin, 2009) and processed by HTSeq (Anders et al., 2015) to get the total gene reads count. Approximately 87% of all reads were mapped back, 95% of which were uniquely mapped to only one location and could be assigned to a single annotated TAIR10 gene. A comparison of the samples shows a very high correlation (Pearson's Correlation Coefficient R^2^>0.92) between wild-type and *fas5* samples (Table S1). Gene counts were normalized and analyzed with the DESeq R package (Anders and Huber, 2010) with a false discovery rate (FDR) of 0.05 as the threshold for differentially expressed genes (DEGs) detection. An enrichment analysis was performed using the DAVID Bioinformatics Resources 6.7 (Huang et al., 2007) and AgriGO Single Enrichment Analysis tool (Du et al., 2010) with an FDR value of 0.05 as the threshold of significance.
